# A rare intraocular lens surface foreign body during phacoemulsification surgery: A case report

**DOI:** 10.1097/MD.0000000000024391

**Published:** 2021-01-22

**Authors:** Chen Li, Peirong Lu

**Affiliations:** Department of Ophthalmology, the First Affiliated Hospital of Soochow University, Suzhou, Jiangsu Province, China.

**Keywords:** case report, intraocular foreign body, intraocular lens, routine phacoemulsification

## Abstract

**Rationale:**

Only a few cases of intraocular lens (IOL) opacification during phacoemulsification surgery have been reported in the literature; intraoperative emergency due to IOL surface foreign body is even rarer.

**Patient concerns:**

A 76-year-old woman underwent uncomplicated cataract surgery in her right eye. A triangular transparent seemingly foreign body tightly attached to the posterior surface of the IOL was found during IOL implantation; the IOL surface foreign body prevented the patient from obtaining satisfactory visual acuity after surgery.

**Diagnosis:**

IOL surface foreign body.

**Interventions:**

After confirmation of the surface foreign body by swept-source optical coherence tomography (IOL Master 700), the surface foreign body was removed in a second surgery. After surgery, the IOL was still well centered.

**Outcomes:**

Fortunately, the patient achieved distinctly improved vision without any visual disturbances in her right eye. To identify the material of the foreign body, it was examined by Fourier-transform infrared spectroscopy (FTIR).

**Lessons:**

This case suggests that surgeons should carefully observe IOLs before implantation. In addition, effective preoperative planning and skillful surgery can remove foreign bodies smoothly and improve patient vision.

## 1 Introduction

Cataract surgery with intraocular lens (IOL) implantation is the most common ophthalmic surgical operation.^[1]^ Since the first IOL implantation in the 1940s, IOL implantation during cataract surgery is a well-established process, and the success rate is much higher than other types of medical foreign material implantations.^[2]^ Due to modern IOL technology and production, IOLs have high biocompatibility and biosafety. Complications or adverse events associated with the IOL itself are uncommon, but have been reported occasionally. One of these situations is IOL opacification during or after surgery, which impacts postoperative visual acuity and requires surgeons to perform further procedures, including posterior capsulectomy, IOL explantation, or even vitrectomy. Most reports of IOL opacification are due to various IOL biomaterials, storage methods, surgical technique, or a combination of these factors.^[3,4]^ To date, there are no reports of IOL surface foreign bodies during cataract surgery.

Here, we report a rare case of an IOL surface foreign body tightly attached to the IOL posterior surface during surgery, which seriously influenced the patient's postoperative visual acuity. After foreign body confirmation by swept-source optical coherence tomography (SS-OCT; IOLmaster700, Carl Zeiss Meditec, Jena, Germany), the foreign body was removed in a second operation. The IOL was well centered, and the patient eventually achieved significantly improved vision.

## 2 Case report

A 76-year-old female was referred to the Eye Center in May 2020 with the chief complaint of vision deterioration for the past several months. Her preoperative corrected distance visual acuity (CDVA) was 20/100 in the right eye and finger counting at 50 cm in the left eye (Snellen chart). Ocular examinations revealed that the ocular surface, pupillary reaction, and intraocular pressure were normal. The fundus examination and macular optical coherence tomography (OCT) revealed a normal right eye but age-related macular degeneration (AMD) in the left eye. Slit-lamp microscopy of the anterior segment showed obvious cataracts (C3N3P5 with LOCSII) in both eyes. An optic biometer (Lenstar, LS 900, Haag-Streit AG, Switzerland) assessment was performed for both eyes. The axial length was significantly longer in the left eye (29.87 mm) compared with the right eye (25.66 mm). The anterior chamber was slightly deeper in the left eye (4.14 mm) compared with the right eye (3.78 mm). The lens thickness was 4.00 mm in the right eye and 3.71 mm in the left eye. Based on these findings and the patient's request, we decided to perform phacoemulsification surgery on her right eye first and implantation of a +15.0 D IOL with -3.41D reserved according to the Barrett formula.

**Figure 1:**
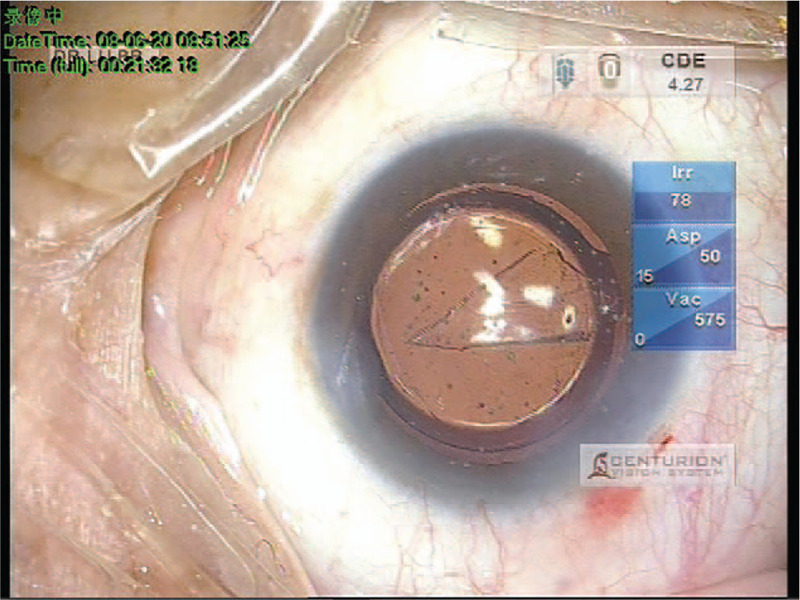
A triangular transparent seemingly foreign body was observed during surgery.

The operation was performed under topical anesthesia. A 2.2 mm self-sealing temporal limbal micro-incision was made at 11 o’clock. A 6.0 mm-diameter capsulorhexis and the phacoemulsification was conducted using the phaco-chop technique. Following phacoemulsification and complete cortical material removal with irrigation/aspiration (I/A) probes, the anterior chamber was filled with viscoelastic agent (IVIZ, Bausch & Lomb, USA). The IOL (Tecnis ZCB00, Johnson & Johnson Vision, USA, SN: 5942861910 with an expiration of October 2023), a foldable, hydrophobic acrylic anterior-aspheric lens with a total diameter of 13 mm and an optic diameter of 6 mm, was injected into the eye. The IOL had been stored at room temperature, and its outer package was intact without any hint of damage. Once the optic region was implanted in the eye, we were surprised to observe a triangular transparent seemingly foreign body on the posterior surface of the IOL (Fig. 1).

The I/A mode was used to insert the tip of the I/A probe into the front and back surfaces of the IOL; the process was continued for a few seconds to allow the foreign body to fully spread. We attempted to remove the foreign body, but the effort seemed futile. Considering the IOL itself was potentially abnormal, we decided to leave it inside the capsular bag and observe the postoperative results prior to applying further removal measures. The procedure was completed by fully rinsing the anterior and posterior chambers and completely removing the viscoelastic agent.

**Figure 2:**
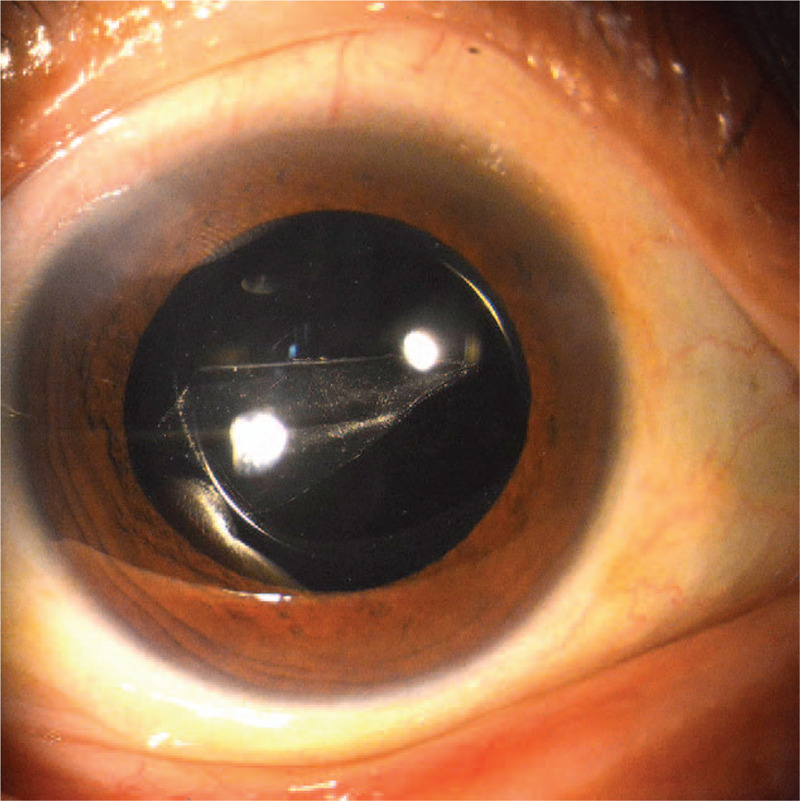
Clinical photograph showing the foreign body on the posterior surface of the intraocular lens.

On the first postoperative day, the patient complained of hazy vision. Her uncorrected distance visual acuity (UDVA) was 20/125, and the CDVA was 20/50 with a manifest refraction of (-3.75) D. The slit-lamp examination showed that the ocular anterior segment was normal and the IOL was well centered. However, the foreign body was tightly adhered to the posterior surface of the IOL (Fig. 2). TobraDex ophthalmic suspension and levofloxacin eye drops were administered 4 times daily in the right eye.

**Figure 3:**
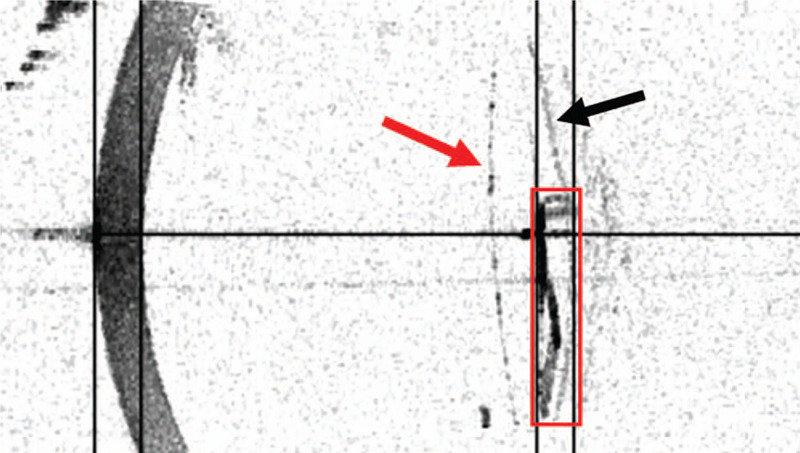
Swept-source optical coherence tomography (SS-OCT; IOLmaster700) showing the foreign body located behind the optical region of the intraocular lens. The red box indicates the foreign body; the red arrow indicates the intraocular lens; the black arrow indicates the posterior capsule.

**Figure 4:**
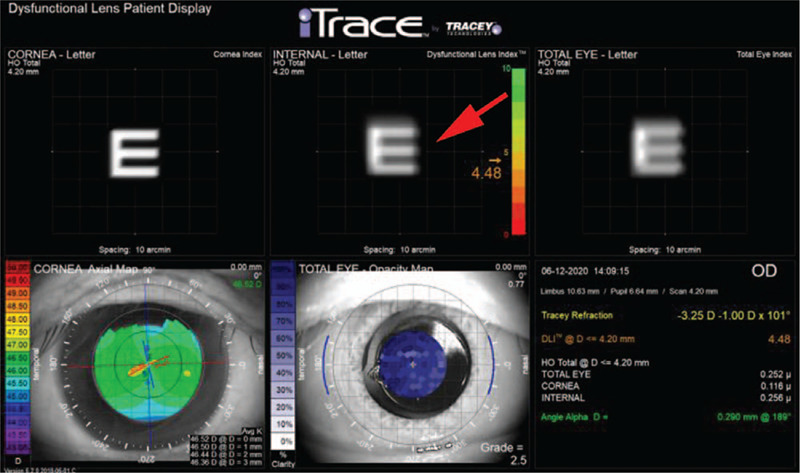
The iTrace aberrometry showing blurred E-chart arising from internal optics (red arrow).

On the fourth day after surgery, we used swept-source optical coherence tomography (SS-OCT) (IOLmaster700, Carl Zeiss Meditec, Jena, Germany) to locate the foreign body. No obvious decentration and transposition of the IOL were detected. The foreign body was located behind the optical region of the IOL; its upper and lower end were fixed to the IOL with a small gap in the middle, suggesting it was an IOL surface body (Fig. 3). Moreover, blurred E-chart arising from internal optics was recorded using the iTrace aberrometry system (Tracey Technologies Corp., Houston, TX) (Fig. 4).

To remove the foreign body and improve the patient's visual acuity, we performed a second operation with the informed consent of the patient. Following routine disinfection and local anesthesia, viscoelastic agent was injected into the anterior chamber and capsular bag through the original main incision. The IOL surface foreign body complex was adjusted to the anterior chamber, and the foreign body was separated and removed by using capsulorhexis forceps. No obvious abnormalities of the intraocular lens back surface were observed. The residual viscoelastic agent was removed by I/A, and the IOL position was reset. The following day after surgery, the patient's UDVA had improved to 20/50 and the CDVA was 20/25 with a manifest refraction of (-3.50) D. The IOL was well centered and no corneal edema was seen. The post-operative therapeutic regimen was antibiotics and corticosteroids eye drops, with a decreasing dosage within 1 month. In follow-up visits over 3 months, the patient's eye was completely normal.

**Figure 5:**
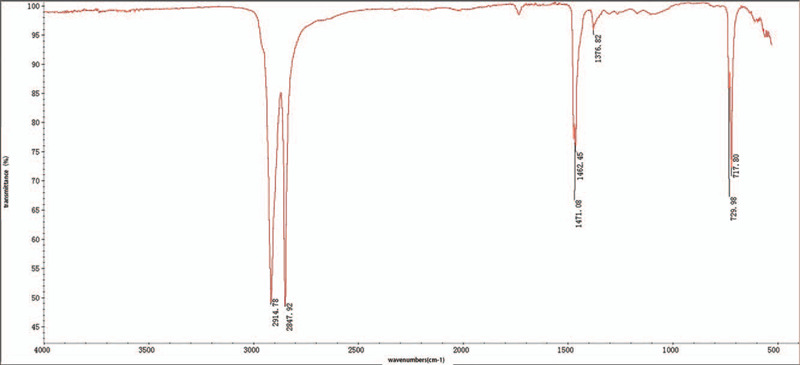
The waveform showing polyethylene as the main component of the foreign body by fourier-transform infrared spectroscopy.

We sent the explanted foreign body to a research center (Microspectrum Chemical Technology Service Co., Ltd, Shanghai, China). The foreign body was evaluated by fourier-transform infrared spectroscopy (FTIR). The results of this evaluation showed it was a polyethylene material (Fig. 5).

The patient read and signed the informed consent for both operations and publication of this case report and related images. The ethics committee of the First Affiliated Hospital of Soochow University approved the current study.

## 3 Discussion

We report an unusual case of an IOL surface foreign body accompanying IOL implantation during surgery, which had a significant effect on visual acuity postoperatively. IOL transient opacification during and after surgery has been reported in previous studies; however, to the best of our knowledge, the implantation of a foreign body attached to IOL has rarely been reported.

Foreign bodies on the surface of the IOL, intraoperatively or postoperatively, are most likely to be attributable to deposits. As an implantable biomaterial, IOL implantation will inevitably lead to a series of biocompatibility responses in patients. In previous reports, the IOL opacification occurred due to the formation of organic deposits on IOL components or the presence of impurities in the polymer. Bang et al^[5]^ reported seven patients who had delayed opacification of the IOL due to calcium deposits. Another experimental study also detected localized calcification of hydrophilic acrylic intraocular lenses after various posterior segment procedures.^[6]^

Opacification of IOLs may also occur after vitrectomy with gas or silicone oil filling; this has led to delayed opacification that occurred several days or even several months after surgery. Marcovich et al^[7]^ and Yamashita et al^[8]^ described opacification of hydrophilic acrylic IOLs following vitrectomy and intravitreous gas injection, and they found calcium aggregate deposits on the anterior surface of the IOL. The possible mechanism was that dehydration may induce chemical alteration of the IOL surface, leading to deposition of calcium and phosphorous from aqueous humor on the exposed areas. In addition, McLoone et al^[9]^ reported deposits on the IOL surface caused by adhesion of silicone oil.

Unlike previous studies, the IOL foreign body in this case was found at the moment of implantation. It is extremely unlikely that such a large deposition formed instantaneously, and after identifying the material as polyethylene, this conjecture was rejected. Given that the main component of the foreign body was polyethylene, it is likely to have come from IOL production or packaging processes; however, the exact source remains unclear.

In past reports, most foreign bodies in the anterior segment come from open global injuries. In these cases, magnets, viscoelastic agent, or micro-instruments were used to move the foreign body to the appropriate position, and forceps were used to remove it through the original wound or incision.^[10]^ In another case, a large silicone oil droplet adhesion on the posterior surface of IOL was removed by using a vitreous cutter.^[11]^ Removal or replacement of the IOL was often adopted in cases where postoperative visual acuity of patients was seriously affected or where complications were caused by IOL decentration, tilt, rotation, or luxation.^[12]^ In this case, the SS-OCT results showed a gap between the foreign body and the posterior surface of the intraocular lens, suggesting that the intraocular lens itself was normal and providing space for us to grasp the foreign body. During surgery, we used capsulorhexis forceps to gently peel the foreign body from the IOL; we removed it smoothly through the original incision without any damage to the posterior surface of the IOL.

The implantation of a foreign body attached to an IOL is a rare adverse event in cataract surgery. Before removing surface foreign bodies, an appropriate plan should be made carefully. The size and location of the foreign body should be evaluated by biometry devices, such as SS-OCT in our case; during operation, the surface foreign body should be gently removed with appropriate micro-instruments to avoid damaging the IOL itself, especially the optical region. Even when strict protocol is followed, surgeons must remain alert to potential errors associated with implantation of IOLs. It is worth mentioning that, in this case, because I/A perfusion could not change the status of the intraocular lens, we made further exploration to distinguish abnormality of the IOL itself and identify the foreign body stuck to the posterior surface of the IOL. It is also essential to thoroughly examine intraocular lenses before implantation, which may greatly reduce the occurrence of unexpected accidents and complications.

This was a rare case where a foreign body was found adhering to the posterior surface of an IOL during surgery. There are few similar reports in the previous literature. This study suggests that surgeons should carefully observe IOLs before implantation. In addition, effective preoperative planning and skillful surgery can remove the foreign body smoothly and improve patient vision.

## Author contributions

**Conceptualization:** Peirong Lu.

**Data curation:** Chen Li, Peirong Lu.

**Formal analysis:** Chen Li.

**Supervision:** Peirong Lu.

**Validation:** Peirong Lu.

**Writing – original draft:** Chen Li.

**Writing – review & editing:** Peirong Lu.
